# Effects of memory cue and interest in remembering and forgetting of gist and details

**DOI:** 10.3389/fpsyg.2023.1244288

**Published:** 2023-12-08

**Authors:** Zhongyu Hu, Jiongjiong Yang

**Affiliations:** School of Psychological and Cognitive Sciences, Beijing Key Laboratory of Behavior and Mental Health, Peking University, Beijing, China

**Keywords:** forgetting, gist and details, memory cue, interest, external and internal

## Abstract

The gist and details of an event are both important for us to establish and maintain episodic memory. On the other hand, episodic memory is influenced by both external and internal factors, such as memory cue and intrinsic motivation. To what extent these factors and their interaction modulate memory and forgetting of gist and detailed information remains unclear. In this study, 29 participants watched film clips accompanied by either gist or detailed cues and rated their interest in these clips. Their memories of gist and detailed information were tested after 10 min, 1 day, and 1 week. The results showed that memory cue modulated the forgetting of gist and detailed memories. Specifically, when gist cues were used, gist memory was forgotten more slowly than detailed memory. When detailed cues were used, detailed memory was forgotten more slowly than gist memory. Differently, the subjective interest in the clips enhanced memory accuracy irrespective of memory type but did not influence the forgetting of gist and detailed memories. Moreover, there was a significant interaction between memory cue and interest, showing that gist cues enhanced memory than detailed cues only for low-interest clips. These results suggest that external and internal factors have differential effects on memory and forgetting, and the effectiveness of external factors depends on the state of intrinsic motivation. The significant interplay of different factors in influencing the remembering or forgetting of gist and detailed memories provides potential ways to enhance memory and retention of gist and detailed information.

## Introduction

When experiencing an episodic event, we not only remember its gist contents (e.g., the central features of the episodes) but also its detailed information (e.g., the perceptual features of the contents; Sekeres et al., 2016, 2018; Robin and Moscovitch, 2017). Both the gist and details that an event contains are important for us in establishing and maintaining episodic memory associated with the experience, connecting the past to the present, but to what extent the two types of memory change over time is debated. It is generally accepted that detailed memory is more subject to decay than gist memory, leading to poorer memory for details over time but better retention of gist information (Frankland and Bontempi, 2005; Furman et al., 2007; Dudai et al., 2015; Sekeres et al., 2016; Zeng et al., 2021). Studies using film clips as materials (e.g., Furman et al., 2007; Leal et al., 2019) and central features as memory cues (e.g., Sekeres et al., 2016) lend support to this view, but in other studies, although performance of gist memory is higher than that of detailed memory, the two types of memory decrease at a similar rate, especially when object images are used as materials (e.g., Talamini and Gorree, 2012; Andermane and Bowers, 2015; Hu et al., 2021). Notably, gist memory was even forgotten more quickly than detailed memory when participants were asked to make detailed descriptions of pictures (e.g., Hu et al., 2021).

One way to reconcile the inconsistent findings is to take the memory cue into account. In our daily lives, various external cues could guide our attention or cognitive resources to different aspects of information about the same episode and influence our memory. For example, we may retain a better memory of a place’s general information (e.g., main routes) or its specific features (e.g., snacks) after we travel to a new city, which is influenced by what a travel brochure or a tour guide has introduced to us. When we talk with friends about traveling, the information we retrieve is influenced by what the friends mentioned, such as a scenic spot or a special food. Memory cues thus are informative enough to function as clues to encode and retrieve specific memory information.

Recent studies have suggested that memory cue during encoding (e.g., Grilli et al., 2019; McCrudden, 2019) and retrieval (e.g., Rudoy et al., 2009; Folville et al., 2020) modulates forgetting of gist and detailed information. In a recent study by Hu et al. (2021), participants were presented with pictures of common objects and were asked to name them or describe their details. The results showed that after the naming task, gist and detailed memories were forgotten at a similar rate, but after the description task, detailed memory was forgotten more slowly than gist memory. These results are consistent with theoretical frameworks that distinguish between different types of memory representations (e.g., Brainerd and Reyna, 1990, 2002; Clark and Paivio, 1991; Moscovitch et al., 2016; Sekeres et al., 2018). For example, the fuzzy-trace theory proposes that a verbatim/specific trace and a gist trace are encoded in parallel, stored separately, and can be retrieved independently of each other (Brainerd and Reyna, 1990, 2002). The trace-transformation theory also proposes that different types of memory (e.g., schematic, gist, and detailed) are separately dependent on different neural bases (Moscovitch et al., 2016; Robin and Moscovitch, 2017). They can co-exist in the brain and be retrieved independently due to task demands. Therefore, the forgetting of these representations could be separately modulated by different tasks or external cues.

Compared with visual images, film clips have distinct storylines (Sonkusare et al., 2019), complex perceptual details, and evoke various subjective feelings, such as humor, interest, or boredom. People usually have a clear memory of a film’s central theme but a less clear memory of its detailed episodes, particularly with the passage of time (Furman et al., 2007, 2012; St-Laurent et al., 2014, 2016; Sekeres et al., 2016; Bonasia et al., 2018). Note that in these studies and daily lives, the titles of the clips that aid in memorizing and recalling specific episodes are typically composed of gist information, such as a main character or basic storylines (St-Laurent et al., 2014, 2016; Sekeres et al., 2016; Bonasia et al., 2018), which could make memory cues more gist-based and thus result in a more sustained gist memory (Sekeres et al., 2016), but in addition to gist cues (e.g., “Godfather”), some detailed cues are also used as film titles (e.g., “Scent of a woman”). These titles not only function as cues to identify specific clips (Sekeres et al., 2016; Bonasia et al., 2018) but also enable participants to attend to different aspects of information when they watch the clips. Therefore, it is helpful to adopt film clips as stimuli (Furman et al., 2007, 2012; Hasson et al., 2008; van Kesteren et al., 2010; Ben-Yakov and Dudai, 2011; Ben-Yakov et al., 2013, 2014; St-Laurent et al., 2014, 2016; Sekeres et al., 2016; Bonasia et al., 2018) to further clarify to what extent memory cues influence forgetting of different types of memory.

In addition to the factors that can be externally manipulated, episodic memory is influenced by internal factors, such as intrinsic motivation. Individuals can spontaneously generate intrinsic drives to explore the event and selectively remember information that is highly motivating, such as clips with high interest. Studies have shown that people tend to remember the events that they are more interested in (Kang et al., 2009; Murayama and Kuhbandner, 2011; Gruber et al., 2014; Fastrich et al., 2018; Fandakova and Gruber, 2021). However, few studies have explored to what extent memory and forgetting of gist and detailed information is modulated by intrinsic motivation. More importantly, intrinsic motivation has distinct characteristics that differ from external factors, which may lead to different impacts on memory and forgetting. First, high intrinsic motivation (e.g., curiosity) enhances subsequent memory, including the memory of answers to trivia questions and of contextual faces (Gruber et al., 2014). Reward promotes memory by capturing attention to reward-associated targets and even irrelevant information (Della Libera and Chelazzi, 2009; Anderson et al., 2011; Gottlieb et al., 2014). Similarly, information with higher intrinsic motivation attracts more attentional resources (Kang et al., 2009; Gottlieb et al., 2014, 2020; Baranes et al., 2015; Wojtowicz and Loewenstein, 2020). Baranes et al. (2015) found that a higher curiosity level led to an earlier anticipatory gaze. Based on these findings, it is reasonable to hypothesize that both gist and detailed memories are enhanced when intrinsic motivation is higher.

Second, interest-motivated memory enhancement persists over time (McGillivray et al., 2015; Fastrich et al., 2018) and is not affected by sleep-dependent consolidation (Stare et al., 2018). For example, in a study by McGillivray et al. (2015), participants recalled the answers 1 h or 1 week after the presentation of trivia questions. The results showed significant effects of post-answer interest on memory for both short and long delays. These findings suggest that intrinsic motivation may not rely on overnight and long-term consolidation but rather on a more rapid learning and consolidation mechanism (Hebscher et al., 2019). Including different time intervals could provide direct evidence to test this assumption.

Furthermore, previous studies have suggested that extrinsic and intrinsic motivations interact to influence subsequent memory. For example, in a study by Murayama and Kuhbandner (2011), two groups of participants learned high- and low-interest trivia questions—one group with monetary rewards and the other without. The results showed that memory enhancement due to monetary rewards was only significant for the low-interest questions. This phenomenon is referred to as the undermining effect in the reward and motivation studies (Deci et al., 1999; Murayama et al., 2010; Murayama and Kuhbandner, 2011; Swirsky et al., 2021), but to what extent a non-reward external factor and intrinsic interest interact to influence memory and forgetting is unclear. When they are included in the same study, due to the fact that attention and cognitive control processing is involved in both the effects of task demand and intrinsic motivation on memory (Murayama et al., 2010; Gruber and Ranganath, 2019; Gottlieb et al., 2014, 2020), they may compete each other for the limited resources; thus, a similar undermining effect appears. Clarifying this issue would help us understand whether there is a general mechanism for the interaction between external and internal impacts on memory.

In sum, the objective of this study was to investigate to what extent the external and internal factors modulated remembering and forgetting of gist and detailed information. To this end, we manipulated the external factor using film clips with gist and detailed cues and measured gist and detailed memories at three different intervals. During encoding, participants watched a series of film clips, half of which were presented following gist cues (e.g., “couple fighting”), and the other half were presented following detailed cues (e.g., “white piano”). The gist cues comprised the central themes of the plots, while the detailed cues captured perceptual details derived from the clips. After delays of 10 min, 1 day, and 1 week, the participants performed forced-choice tests with three-option questions to measure their gist and detailed memory. The level of interest was subjectively evaluated after the participants viewed each of the film clips. The forgetting rates were calculated by assessing the percent difference between accuracy at 1 week and that at the immediate test. This design allowed us to examine how non-motivational external factors and intrinsic interest interact to affect both gist and detailed memory and forgetting.

We hypothesized that factors of memory cue and subjective interest in film clips would have different effects on memory and forgetting of gist and detailed information. Specifically, gist cues would decrease the forgetting of gist memory, whereas detailed cues decrease the forgetting of detailed memory. Although interest enhances memory performance, it would not vary in memory type and influence forgetting over time. Furthermore, the external and internal factors interact to influence subsequent memory. The effect of memory cue on accuracy would be diminished when the process is more internally motivated.

## Materials and methods

### Participants

To achieve a power of 0.95 with a medium effect size of *f* = 0.25 and *α* = 0.05, an *a priori* power analysis using G*Power 3.1 (Faul et al., 2009) for within factors ANOVA of 2 (memory cue) * 2 (memory type) * 3 (retention interval) indicated that a sample size of 22 participants would be required (the value of non-sphericity correction was set to 0.75). A similar power analysis of 2 (memory cue) * 2 (memory type) * 2 (interest) * 3 (retention interval) measurement was also conducted, and a sample size of 14 was obtained. Because of the smaller number of clips (36 in total), to diminish the possibility of insufficient power, a total of 30 participants (nine male participants, with a mean age of 21.90 ± 2.64 years) were included in the experiment. All participants were right-handed and fluent in Chinese with no history of psychiatric or neurological disorders. The procedures were approved by the ethics committee of the School of Psychological and Cognitive Sciences, Peking University. All participants gave their written informed consent and were paid with money or credits.

### Materials

Four factors were included in the study: memory type (gist, detailed), memory cue (gist, detailed), interest (low, high), and retention interval (10 min, 1 day, and 1 week). All of them were treated as within-subject factors. Among them, the levels of interest were determined individually by self-report ratings.

Thirty-six film clips were used as materials in the experiment. Among them, 22 clips were selected from previous studies (St-Laurent et al., 2014, 2016; Sekeres et al., 2016), and 14 clips were selected from the Internet. Each of the clips was around 24 s in duration (24.61 ± 7.94 s) and included a coherent plot of human activities and limited dialogue. The perceptual features of the film clips were evaluated by an independent group of participants (*N*_1_ = 24; 12 male participants, with a mean age of 22.29 ± 2.80 years). These features included visual complexity (color complexity, background complexity, and movement complexity), story complexity (storyline complexity and character complexity), sound complexity (dialogue complexity, background music complexity, and noise complexity), and emotional features (valence and arousal; 1–5 for all ratings, 1 indicating the least and 5 indicating the most; Sekeres et al., 2016). The selected film clips had medium visual complexity (2.89 ± 0.32), story complexity (2.53 ± 0.37), sound complexity (2.32 ± 0.29), and emotional levels (valence 3.08 ± 0.58 and arousal 2.49 ± 0.37).

The gist and detailed memory cues were obtained based on a pilot study. A separate group of participants (*N*_2_ = 10; nine male participants, with a mean age of 22.20 ± 2.10 years) watched a total of the 73 film clips, summarized the central storylines, and recalled the gist and detailed information as much as possible. The gist (3.36 ± 1.31 words) and detailed (4.42 ± 0.81 words) memory cues were determined based on the participants’ responses and the experimenters’ evaluations. The gist cues summarized the main storylines or main characters of the episodes (e.g., “couple fighting” and “eating dessert”), while the detailed cues were derived from perceptual information in the episodes (e.g., “white piano” and “white ice cream scoop”; Table 1). The questions used to test gist and detailed memories were also selected by the same pilot study. According to the descriptions of the participants, gist questions (number as 4.78 ± 0.59) were created to test the main storyline of each clip, e.g., what happened and who did it. Detailed questions (number as 5.22 ± 0.59) were created to test the perceptual information, e.g., the appearance of a character and the color of an object (Sekeres et al., 2016; Table 1). For each question, three answer options were provided, with one as the correct answer and the other two as foils that were falsely reported by the participants. The information mentioned in the memory cues was not tested during the forced-choice tasks.

**Table 1:** Examples of the stimuli.

Gist cue	Detailed cue	Gist question	Option (*Correct Answer*)	Detailed question	Option (*Correct Answer*)
Couple fighting	White piano	Besides the couple, who else is the main character in the clip?	A. A little boy	What color sweater is the little girl wearing?	A. White
			B. A nanny		B. Yellow
			*C. A little girl*		*C. Pink*
		What are the couple doing?	A. Doing housework	What does the little girl’s hair look like?	A. Short black hair
			*B. Arguing*		*B. Straight black shawl*
			C. Cooking		C. Black ponytail
		What is the girl doing?	A. Doing homework	What is the status of the room door?	A. Wide open
			B. Watching TV		B. Closed
			*C. Playing the piano*		*C. Half open*
		How does the girl play the piano?	*A. Very irritable*	What does mom’s hair look like?	*A. Short black curls*
			B. Very gentle		B. Black updo
			C. Very smooth		C. Black shoulder-length hair
		Who grabs the girl?	*A. Father*	Which body part of the girl is grabbed?	A. Left shoulder
			B. Mother		*B. Left hand*
			C. Nobody		C. Right hand
Eating dessert	White ice-cream scoop	Who is sitting at the dining table?	A. Dad and son	What is the style of the table?	A. Wooden round table
			B. Mom and son		B. White long table
			*C. Dad, mom, and son*		*C. Black long table*
		What does the boy eat?	*A. Fruit*	What color sweater is mom wearing?	A. White
			B. Ice cream		*B. Red*
			C. Biscuit		C. Blue
		What does the boy not eat?	A. Fruit	How many scoops of ice cream are there?	A. 1
			B. Fruit and ice-cream		*B. 2*
			*C. Ice-cream*		C. 3
		How do the parents react after they see the boy’s behavior?	*A. Surprised*	What does the boy use to eat the fruit?	*A. Spoon*
			B. Relieved		B. Fork
			C. Calm		C. Hands
		Finally, who scolds the boy?	A. Dad	What are the positions of dad, mom, and son at the dining table?	A. Right, middle, and left
			*B. Mom*		*B. Right, left, and middle*
			C. Nobody		C. Left, right, and middle

All materials were presented in Chinese and are translated into English for illustration purpose.

To obtain a baseline performance of the questions (i.e., the probability of accurately answering questions without viewing the film clips), an independent group of participants (*N*_3_ = 16, seven male participants, with a mean age of 21.13 ± 1.93 years) completed a baseline test. The participants were presented with each of the clip titles and questions with three options and were asked to choose one option. Because they did not watch the film clips, they were encouraged to answer the questions by guessing. The results showed that the baseline performance (*Mean* = 0.33, *SD* = 0.06) was at the chance level under each condition (*p*’s > 0.30). A 2 (memory cue) * 2 (memory type) ANOVA for accuracy revealed that the main effects and their interaction were not significant (*p*’s > 0.10). It indicated that without watching the film clips, the accuracy was well-controlled to a chance level and comparable for different memory cues and memory types.

The clips were divided into six sets and used for two types of memory cues and three retention intervals. For each set, there were six film clips, with about 30 gist questions and 30 detailed questions. The features of film clips (visual complexity, story complexity, sound complexity, and emotional content), the words of the gist and detailed memory cues, and the baseline performance of the gist and detailed questions were matched among all the sets (*p*’s > 0.05). The clips were counterbalanced across the participants to ensure that all the clips had an equal chance to be used under different conditions.

### Procedures

**Figure 1:**
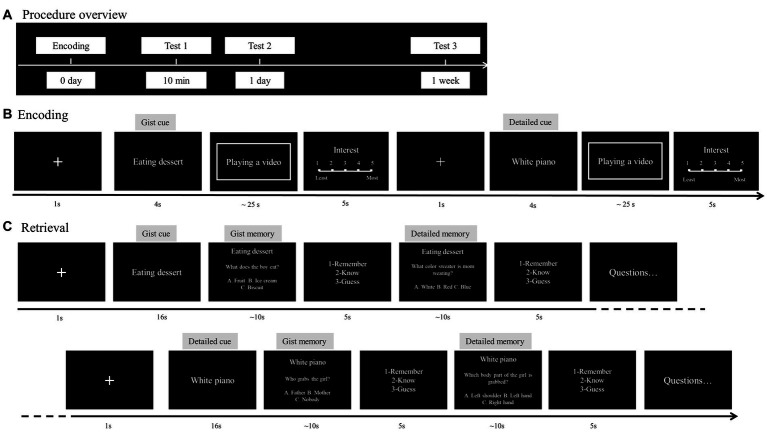
Procedures of the experiment. **(A)** The procedure consists of an encoding and three retrieval phases. **(B)** During encoding, a memory cue was presented for 4 s. Then, a film clip was presented with sounds. After watching the clip, the participants rated their interest in this clip. **(C)** During retrieval, the memory cue was presented for 16 s. The participants answered the forced-choice questions followed by RKG judgments. The memory cues and questions were presented in Chinese and are translated into English for illustration purpose.

The procedure included one encoding phase and three retrieval phases (Figure 1A). The participants learned the 36 film clips on the same day and then took memory tests 10 min, 1 day, and 1 week later.

During the encoding phase, the memory cue of a film clip was first presented in the center of the screen for 4 s (Figure 1B). The participants were told that the cue was used for the same clip during the encoding and retrieval phases. Then, the clip was presented, and the participants were asked to remember the film clip’s content as much as possible. When the clip disappeared, the participants rated their interest in the film clip (i.e., the level of willingness they had to continue to watch the clip; 1 = *least interested*, 5 = *most interested*). Half of the film clips were presented with gist cues (e.g., “coupling fighting”) and the other half with detailed cues (e.g., “white piano”). The 36 film clips were randomly presented during encoding so that no more than three clips belonging to the same conditions appeared consecutively.

During each retrieval phase, the participants performed a forced-choice task to test their gist and detailed memories (Figure 1C). Similar to studies of autobiographical memory and studies using film clips as stimuli (Sekeres et al., 2016; Hebscher et al., 2018; McCormick et al., 2020), the memory cue of the tested clip was first presented for 16 s, during which the participants were asked to visualize the clip in their mind from beginning to end. This manipulation enabled the participants to construct the story contents and elaborate perceptual details as much as possible. Next, the gist and detailed questions for the same clip were randomly presented in a block. The participants were asked to choose the correct answer from three options (up to 10 s) and make a remember/know/guess judgment afterward. If they were confident about the answer and were able to vividly retrieve the related contents, they responded with “remember.” If they were not very confident about the answer and forgot the specific episodes, they responded with “know.” Otherwise, they responded as “guess.” The memory cue and questions for the next clip were presented after all the questions for the previous clip had been answered. The 12 clips for each retrieval phase were tested in a random order, with half of them previously encoded with gist cues and the other half encoded with detailed cues.

Before the retrieval phase at the 10-min interval, the participants were asked to count backward by 7 continuously from 1,000 for 5 min as the distractor task, to avoid a rehearsal effect from the encoding phase. The participants had separate opportunities to practice encoding and retrieval trials before the formal phases.

### Statistical analyses

The participants rated their interest levels after they viewed each of the film clips. So, each participant’s trials were split into high and low conditions (15 questions in each) according to that participant’s median curiosity level (e.g., Kang et al., 2009; Halamish et al., 2019) for each memory cue at each retention interval. In a post-hoc analysis, an ANOVA of memory cue * retention interval revealed no significant main effects or their interaction for the medium interest levels (*p*’s > 0.10). It indicates that the cutoff values for the high- and low-interest conditions were comparable in different memory cue and retention interval conditions. Then, the accuracy of the forced-choice task was analyzed using a repeated-measures ANOVA, with memory cue (gist, detail), memory type (gist, detail), interest (high, low), and retention interval (10 min, 1 day, and 1 week) as within-subject factors.

The forgetting patterns of gist and detailed memories were estimated by the interaction between memory type and retention interval (Slamecka and McElree, 1983; Gardiner and Java, 1991; Hockley and Consoli, 1999; Wixted, 2004). In addition, to exclude the influence of initial performance (i.e., memory accuracy at the 10-min interval) on forgetting patterns, the forgetting rate (Fr) was calculated as follows (Murty et al., 2019; Li et al., 2020):


$$\mathrm{Forgeting rate}=\frac{\mathrm{accurac}y_{10-\min}–\mathrm{accurac}y_{1-\mathrm{week}}\;}{\mathrm{accurac}y_{10-\min}}$$

To investigate the influence of memory cue and interest on the forgetting of gist and details, the forgetting rates at 1 week were analyzed by repeated-measures ANOVA, with memory cue, memory type, and interest as within-subject factors. One participant was excluded due to poor memory performance, resulting in 29 participants in the analysis. Partial eta squared (*η*^2^) was calculated to estimate the effect size of each analysis. Post-hoc pairwise comparisons were Bonferroni-corrected (*p* < 0.05, two-tailed).

## Results

### Accuracy

**Figure 2:**
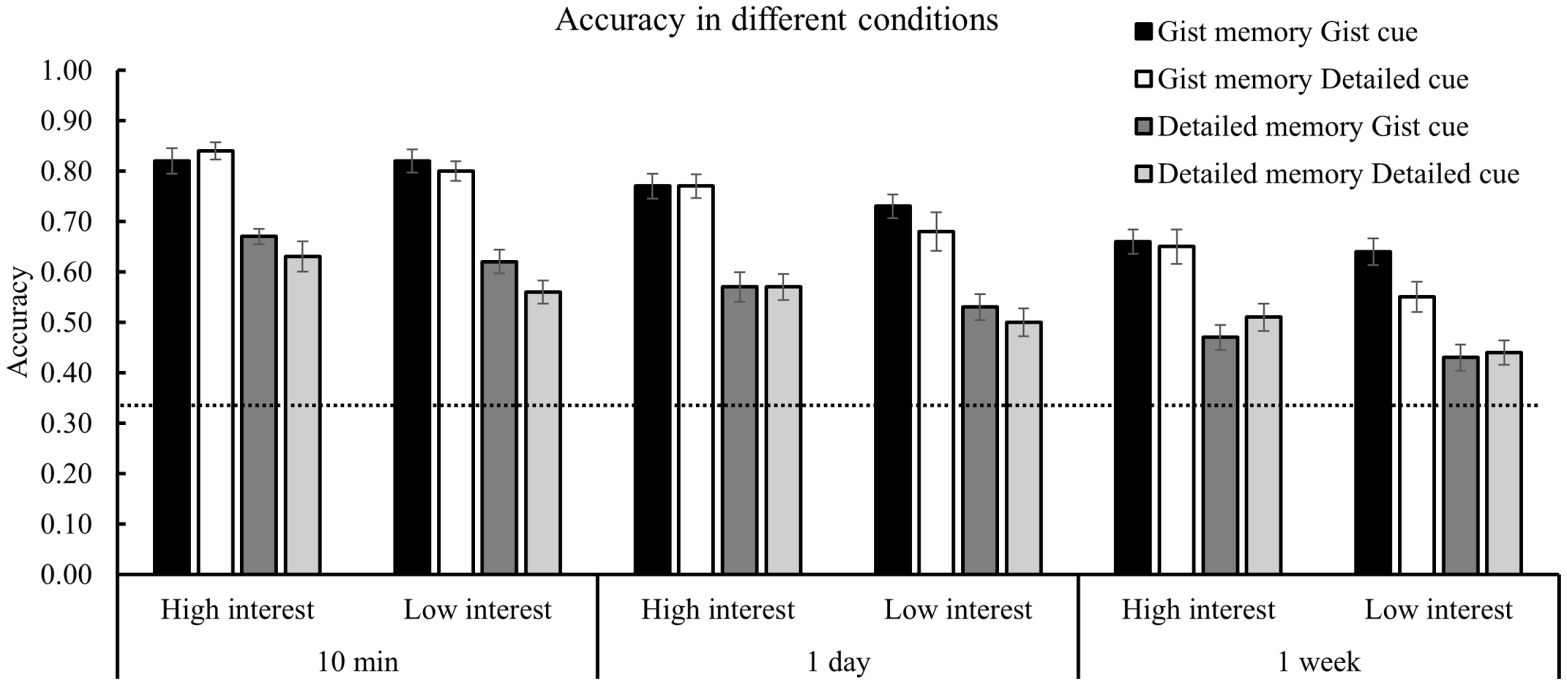
Results of the accuracy in different conditions. There were significant main effects of memory cue, memory type, interest, and retention interval, a significant two-way interaction between memory cue and interest, and a significant three-way interaction among memory cue, memory type, and retention interval.

For the accuracy of forced-choice task (Table 2; Figure 2), the ANOVA of 2 (memory type) *2 (memory cue) * 2 (interest) * 3 (retention interval; Table 3) revealed significant effects of memory cue [*F*(1, 28) = 4.67, *p* = 0.04, *η^2^* = 0.14], interest [*F* (1, 28) = 38.63, *p* < 0.001, *η*^2^ = 0.58], and memory type [*F* (1, 28) = 434.62, *p* < 0.001, *η^2^* = 0.94]. The participants had higher accuracy for the clips with gist than detailed cues and for those with higher than lower interest. The gist questions were answered with higher accuracy than detailed questions. Memory performance declined significantly over time [*F* (2, 56) = 63.91, *p* < 0.001, *η*^2^ = 0.71].

**Table 2:** Accuracy of gist and detailed memories in different conditions.

			10 min	1 day	1 week
Gist memory	High interest	Gist cue	0.82 ± 0.14	0.77 ± 0.13	0.66 ± 0.13
		Detailed cue	0.84 ± 0.09	0.77 ± 0.13	0.65 ± 0.18
	Low interest	Gist cue	0.82 ± 0.12	0.73 ± 0.12	0.64 ± 0.14
		Detailed cue	0.80 ± 0.10	0.68 ± 0.21	0.55 ± 0.16
Detailed memory	High interest	Gist cue	0.67 ± 0.08	0.57 ± 0.16	0.47 ± 0.13
		Detailed cue	0.63 ± 0.16	0.57 ± 0.14	0.51 ± 0.14
	Low interest	Gist cue	0.62 ± 0.13	0.53 ± 0.14	0.43 ± 0.14
		Detailed cue	0.56 ± 0.13	0.50 ± 0.15	0.44 ± 0.13

**Table 3:** SPSS results of the accuracy.

*Source*	*F*	*Sig.*	*η^2^*
Memory cue	4.670	**0.039**	0.143
Interest	38.633	**0.000**	0.580
Memory type	434.624	**0.000**	0.939
Retention interval	68.913	**0.000**	0.711
Memory cue * Interest	7.823	**0.009**	0.218
Memory cue * Memory type	0.377	0.544	0.013
Memory cue * Retention interval	0.156	0.856	0.006
Interest * Memory type	0.244	0.625	0.009
Interest * Retention interval	0.399	0.673	0.014
Memory type * Retention interval	2.139	0.127	0.071
Memory cue * Interest * Memory type	0.907	0.349	0.031
Memory cue * Memory type * Retention interval	4.303	**0.018**	0.133
Memory cue * Interest * Retention interval	0.171	0.843	0.006
Interest * Memory type * Retention interval	0.397	0.674	0.014
Memory cue * Interest * Memory type * Retention interval	0.055	0.947	0.002

The bold values mean that the effects are significant.

**Figure 3:**
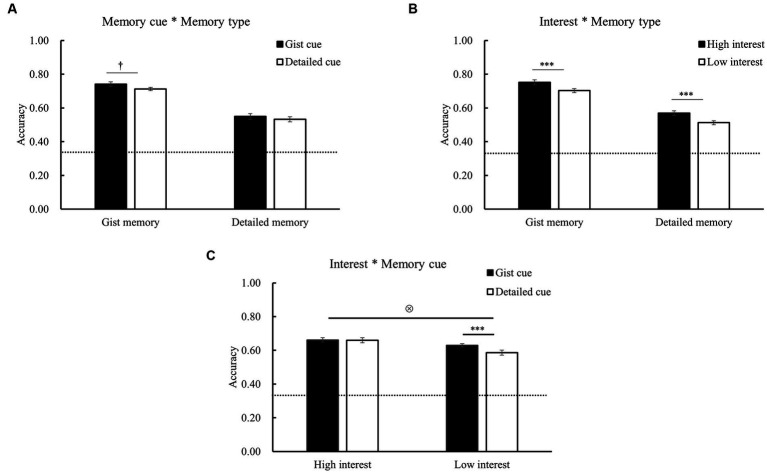
Two-way interaction effects for memory accuracy. **(A)** The participants had higher accuracy for the clips with gist than detailed cues. The interaction of memory cue and memory type was not significant. **(B)** The participants had higher accuracy for the clips with higher than lower interest. The interaction of interest and memory type was not significant. **(C)** There was a significant interaction of interest and memory cue for accuracy. For the clips with low interest, the accuracy was significantly higher when the gist (vs. detailed) cues were used. The error bars represent the standard errors of the means. ^*^p < 0.05, ^***^p < 0.001, and ^+^*p* < 0.10, ⊗ significant interaction.

The two-way interaction between memory cue and memory type [*F* (1, 28) = 0.38, *p* = 0.54] was not significant (Figure 3A). Although gist memory accuracy was higher when gist (vs. detailed) cues were used, the difference did not reach significance (*p* = 0.08). More importantly, there was a significant three-way interaction among memory cue, memory type, and retention interval for accuracy [*F* (2, 56) = 4.30, *p* = 0.02, *η^2^* = 0.13; Table 3]. It indicated that memory cues modulated the forgetting patterns of gist and detailed memories differently, which was confirmed and further clarified by the results of the forgetting rate (shown in the next section).

The internal factor of interest did not show significant interaction with memory type [*F* (1, 28) = 0.24, *p* = 0.63] and interval [*F* (2, 56) = 0.67, *p* = 0.40; Table 3; Figure 3B]. Thus, the interest-related memory enhancement was not affected by memory type or retention interval. Importantly, there was a significant interaction between memory cue and interest [*F* (1, 29) = 6.55, *p* = 0.016, *η^2^* = 0.18; Figure 3C]. Further analysis showed that for the clips with high interest, the effect of memory cue on accuracy was not significant (*p* = 0.89). However, for the clips with low interest, the accuracy was significantly higher for those with gist cues than with the detailed cues (*p* = 0.001). As predicted, an undermining effect was observed between external and internal factors. There were no other significant interest-related interactions (*p*’s > 0.20).

### Forgetting rate

**Figure 4:**
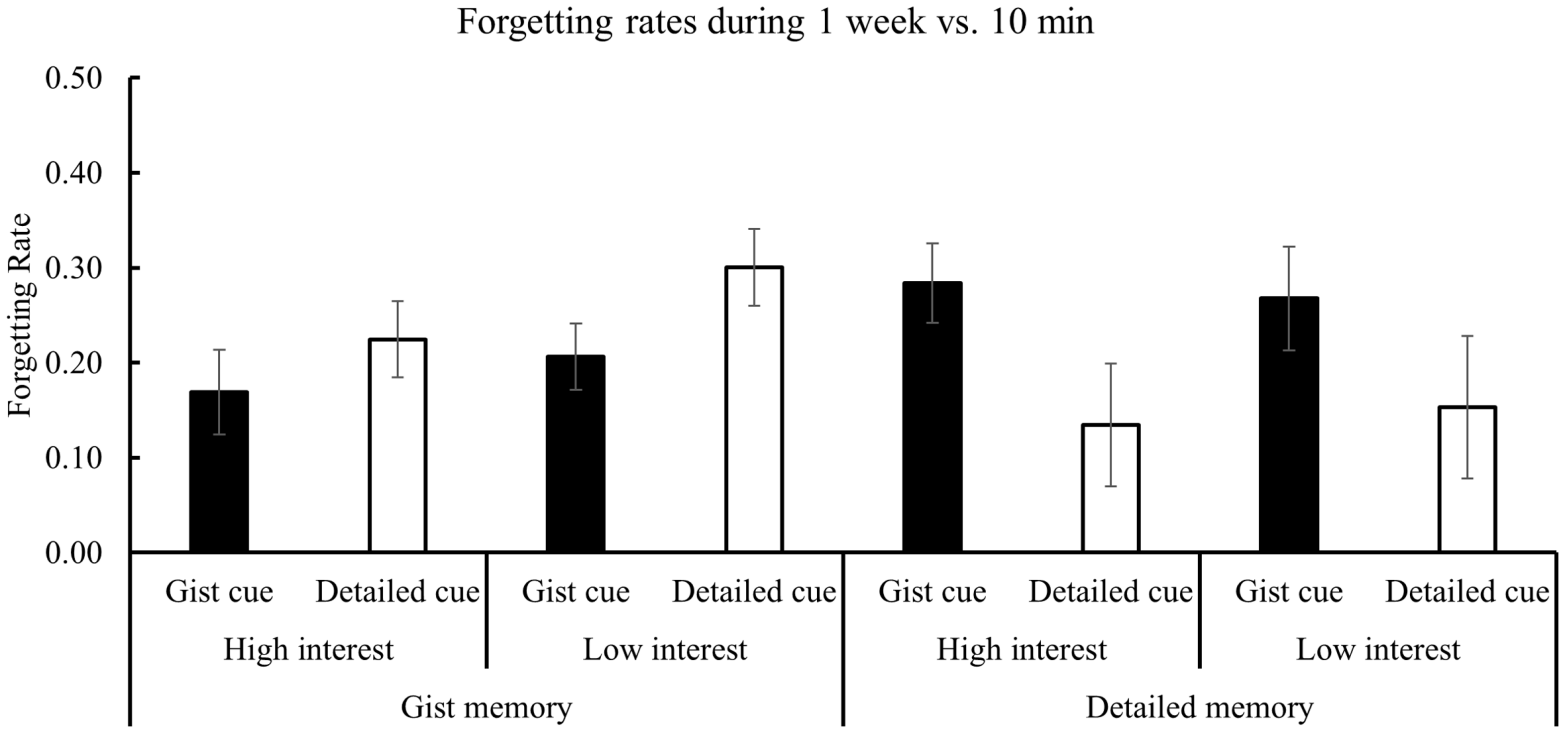
Results of the forgetting rate (1 week vs. 10 min) in different conditions. There was a significant interaction between memory cue and memory type.

**Figure 5:**
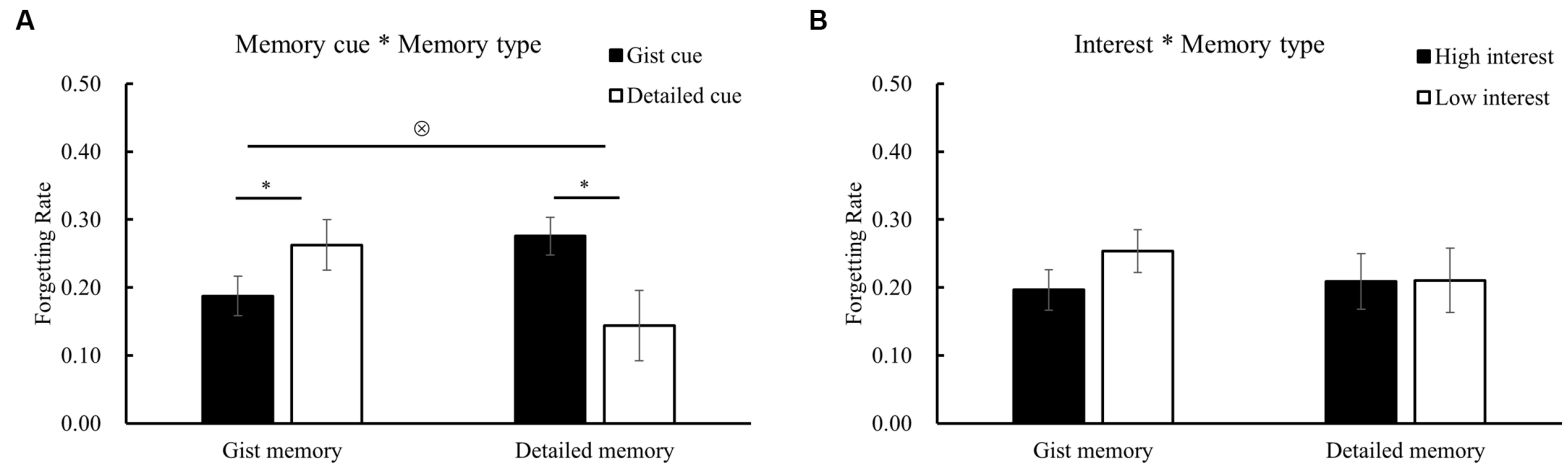
Two-way interaction effects for the forgetting rate (1 week vs. 10 min). **(A)** There was a significant interaction of memory cue and memory type for the forgetting rate. Gist memory was forgotten less when the gist (vs. detail) cues were used, while detailed memory was forgotten less when the detailed (vs. gist) cues were used. **(B)** There was no significant interaction of interest and memory type for the forgetting rate. The error bars represent the standard errors of the means. ^*^p < 0.05, ⊗ significant interaction.

In this study, there was a significant difference between gist memory and detailed memory at 10 min (0.82 ± 0.08 vs. 0.62 ± 0.07, *t*_28_ = 14.38, *p* < 0.001, *Cohen’s d* = 2.67). So, it is better to consider the initial memory difference in analyzing the forgetting pattern. The ANOVA of memory cue * memory type * interest (Table 4; Figure 4) for the forgetting rate revealed a significant interaction between memory cue and memory type [*F* (1, 28) = 8.71, *p* = 0.006, *η^2^* = 0.24; Figure 5A]. Further analyses showed that the forgetting rate of gist memory was significantly slower when the gist (vs. detail) cues were used (*p* = 0.05), while the forgetting rate of detailed memory was reduced when the detailed (vs. gist) cues were used (*p* = 0.04). These results suggest a double dissociated effect of memory cues on the forgetting of gist and detailed memories. Consistent with the results of accuracy, none of the effects and interactions involving interest were significant (*F’*s < 0.80, *p*’s > 0.30). Particularly, the interaction of interest and memory type [*F* (1, 28) = 0.47, *p* = 0.50] was not significant (Figure 5B), which indicated that subjective interest did not influence the forgetting rate of different memory types.

**Table 4:** SPSS results of the forgetting rates.

*Source*	*F*	*Sig.*	*η^2^*
Memory cue	0.618	0.438	0.022
Interest	0.874	0.358	0.030
Memory type	0.178	0.676	0.006
Memory cue * Interest	0.327	0.572	0.012
Memory cue * Memory type	8.709	**0.006**	0.237
Interest * Memory type	0.468	0.500	0.016
Memory cue * Interest * Memory type	0.001	0.980	0.000

The bold values mean that the effects are significant.

We also performed the correlational analysis between the forgetting rate of gist and detailed memories. The results showed that they were not significantly correlated (*r* = 0.19, *p* = 0.33). When the forgetting rates were separately analyzed, the correlations were not significant either under the gist cues (*r* = 0.07, *p* = 0.73) or detailed cues (*r* = −0.11, *p* = 0.55) conditions. It suggests that the forgetting processes for gist and detailed memory are relatively independent of each other.

## Discussion

The primary aim of this study was to investigate to what extent the external factor and the internal factor modulated memory and forgetting of gist and detailed information. Memory cue was manipulated as a within-subject factor, and subjective interest was rated individually. There were three main findings. First, memory cues had double dissociated effects on the forgetting of gist and detailed memory. Gist memory was forgotten less over time than detailed memory when the gist cues were used, whereas detailed memory was forgotten less than gist memory when the detailed cues were used. Second, subjective interest in the film clips improved both gist and detailed memory, but it did not modulate their forgetting rates. Third, the external and internal factors interact to modulate subsequent memory. Only when the clips were less interesting to the participants, memory accuracy was higher for clips with gist (vs. detailed) cues. These results shed light on the different dependence of the external factor and the internal factor on long-term retention, as well as the undermining effect of internal factor on external factor in enhancing subsequent memory regardless of memory type.

### Memory cue and forgetting of gist and details

One novel finding of this study was a double dissociated effect of memory cue on the forgetting of gist and detailed memories. It is intensively debated whether gist and detailed information is forgotten differently over time. Our study clarified this issue by manipulating the memory cue as either gist or detailed types. We found that when gist cues were used, the gist information of the film clips was forgotten more slowly than the detailed information. This was consistent with previous findings, in which gist was retained better than details over days (Sekeres et al., 2016) and months (Furman et al., 2007, 2012) when the memory cues were gist-based. More importantly, our study showed that when detailed cues were used, detailed information was forgotten more slowly than gist information. This pattern occurred after 1 week, indicating a long-term effect of memory cues that help to selectively consolidate cue-related information after encoding. Combined with similar dissociations when object images were used as stimuli (e.g., Hu et al., 2021), these results implicated the important role of memory cues in modulating long-term retention of gist and detailed information. Different memory cues could modulate forgetting of different types of memory and help people more stably consolidate specific information.

Our results were obtained when some confounding factors were well-controlled. First, the baseline performance for gist and detailed memories was controlled at a chance level (0.33), which was obtained from an independent group of participants who did not watch the film clips. Particularly, the gist and detailed questions were answered at a comparable level. This ensured that the difference in memory performance was mainly due to the encoding phase. Second, the relative number of gist and detailed information was controlled in the forced-choice task. In most studies that used film clips or passages, recall tasks were often employed (e.g., van Kesteren et al., 2010; Sekeres et al., 2016). The number of recalled details usually outweighs that of gist at the immediate test, making it easier for detailed memory to show a steep decline. Thus, the forgetting patterns would be distorted (Hasson et al., 2008; Andermane and Bowers, 2015; Leal et al., 2019). Our study created gist and detailed questions and used forced-choice test to diminish the confound. Third, the forgetting rate was estimated as a percent change rather than the accuracy difference between 1 week and 10 min. The forgetting rate is regarded as a behavioral marker for memory consolidation (Litman and Davachi, 2008; Murty et al., 2019). Although the number of questions and the baseline memory performance were matched for gist and detailed memories, gist memory accuracy was significantly higher than detailed memory at 10-min interval. So, it is necessary to control for this initial memory performance (Murty et al., 2019; Li et al., 2020) to better assess the forgetting pattern over time. This measure thus enabled us to assess to what extent memory cue and interest modulated long-term consolidation of gist and detailed memories.

The principle of double dissociation has been widely used. In the field of neuropsychological field, double dissociations offer strong evidence that two patient groups have selective deficits in two cognitive operations. Similarly, a double dissociation can identify whether two cognitive functions are independent of each other (Gazzaniga et al., 2018). The double dissociation of the forgetting of gist and detailed memory in our study thus indicates that forgetting of gist memory and detailed memory is separately modulated by different memory cues. On the other hand, Dunn and Kirsner (1988, 2003) pointed out that the inferential logic associated with double dissociation has some flaws. The double dissociation may still be influenced by a common process/function, but our results showed that the forgetting of gist and detailed memories was not significantly correlated, which suggests that the forgetting processes for gist and detailed memory are relatively independent of each other. Nevertheless, we should be cautious to overinterpret the results of double dissociation. Future neuroimaging studies could lend additional evidence for the functional dissociation between two different processes.

In addition to the forgetting rate, memory cue did not significantly modulate the accuracy of gist and detailed information. It is different from the results of Hu et al. (2021), in which they found that gist memory was significantly higher after the naming (vs. description) task. Although there was a trend that gist memory was enhanced by gist (vs. detailed) cues in this study, the difference was not significant. It is possible that participants preferred to process the central theme of the clips, which leads to higher memory performance of gist information than detailed information irrespective of memory cues. This explanation is supported by the predictions of the fuzzy-trace theory, which proposes that information that is vital to an event (e.g., gist) takes priority in being preferentially processed (Brainerd and Reyna, 1990, 2002). On the other hand, similarly to the current findings, the results of Hu et al. (2021) also showed that detailed memory was comparable after the naming and the detail description tasks. It seems that the description task or detailed cues alone may not be sufficient to enhance the memory performance of perceptual information. Only when detailed information associated with object/event contexts is strongly emphasized, could the detailed memory be significantly enhanced (Grilli et al., 2019).

### Subjective interest and memory of gist and details

Different from memory cue, interest as an internal factor did not influence forgetting. However, it interacted with memory cue to enhance subsequent memory regardless of memory type. Interest is a powerful motivational variable that is characterized as a positive and rewarding feeling toward the knowledge and value of a specific event (Fastrich et al., 2018). In our study, the inherent interest level based on individual ratings ensures that the interest level matches the internalized value. The results showed that memory was enhanced for clips with higher (vs. lower) interest levels, irrespective of gist and detailed information. Similarly, high-curiosity states enhanced memory for both trivia questions and incidental neutral faces (e.g., Gruber et al., 2014). These results are consistent with the assumption of the generalization of reward value on memory (Kang et al., 2009; Gottlieb et al., 2014, 2020; Baranes et al., 2015; Wojtowicz and Loewenstein, 2020). Intrinsic motivation has a priority to activate the attentional system for processing information with higher psychological needs, which is supported by the findings of the co-activation of the anterior hippocampus and dopamine system, as well as the eye movement patterns (Kang et al., 2009; Gottlieb et al., 2014, 2020; Baranes et al., 2015; Wojtowicz and Loewenstein, 2020). Interest could broaden attention to the entire events, trigger proactive exploration and information-seeking behaviors, lead to more in-depth learning and fine-grained memory, and even promote sustainable information acquisition through a positive feedback loop (Murayama, 2022). This leads to memory enhancement of both the gist and detailed components of an episode due to intrinsic motivation.

Although previous study has shown that sleep did not influence the effect of curiosity on the memory of trivia questions (Stare et al., 2018), few studies have directly compared memory at different intervals to track forgetting over time, especially for different types of memories. In this study, by including three retention intervals, we found that memory performance was higher for the high (vs. low) interest condition immediately after encoding and this effect persisted over the 1-week period, but interest level did not influence the forgetting of gist and details. Our findings complement previous studies and indicate that intrinsic motivation could promote memory through encoding activities, with a weak dependence on over-night and long-term memory consolidation (Stare et al., 2018).

Another novel finding of our study was that subjective interest interacted with memory cues to modulate subsequent memory. When the clips were interesting, the effect of interest on memory was dominant, such that gist and detailed cues had similar effects on later memory. However, when the interest level was low, gist cues took the responsibility to help the participants memorize the events (Tullis and Finley, 2018, 2021). These results demonstrated an undermining effect when a non-reward external factor and intrinsic interest were included in the same study. Similarly, when the monetary reward was manipulated and the trivia questions were classified as high and low interesting, Murayama and Kuhbandner (2011) showed that memory enhancement due to reward was only significant when the materials were of low interest. As memory cues and monetary rewards are external factors, both lines of evidence suggest that external and internal factors may interact to modulate memory performance and exhibit an undermining effect (Murayama and Kuhbandner, 2011; Swirsky et al., 2021). This effect is mainly explained by the fact that cognitive control is important for processing information related to task demand and intrinsic motivation (Gottlieb et al., 2014, 2020; Gruber and Ranganath, 2019; Murayama, 2022), so interest and task demands can compete for domination in affecting memory performance.

### Theoretical and practical implications

How different types of memories are formed and retained over time is one of the central issues in memory research. Clarifying the forgetting of gist and detailed memory is important to understand how different types of memory representations change over time. Memory theories distinguish between different levels of representations of episodic memory. Some of them focus on behavioral and cognitive processes (e.g., Brainerd and Reyna, 1990, 2002), whereas others link distinct levels of representations to underlying neural correlates (e.g., Kirwan and Stark, 2007; Yassa et al., 2011; Moscovitch et al., 2016; Robin and Moscovitch, 2017; Andermane et al., 2021). Based on frameworks such as the fuzzy-trace theory and the trace-transformation theory, gist and detailed memories could be separately influenced by some factors, but empirical evidence to identify these factors is still lacking and currently inconsistent. Our study highlighted the important role of memory cues in selectively modulating the forgetting of gist and detailed memories. These findings provided clear evidence for the co-existence of parallel gist and detailed representations of the same episodes as proposed in memory theories (e.g., Brainerd and Reyna, 1990, 2002; Clark and Paivio, 1991; Robin and Moscovitch, 2017; Sekeres et al., 2018).

Moreover, people have a spontaneous tendency to seek out novelty and new information (Ryan and Deci, 2000). So, in many cases, task demands and intrinsic motivation co-exist and dynamically influence goal-directed behavior (Di Domenico and Ryan, 2017). Memory theories have mainly focused on how memory representations are transformed, selectively consolidated, and integrated by the hippocampus, cortical regions, and their interactions (for reviews, Moscovitch et al., 2016; Sekeres et al., 2018; Hebscher et al., 2019; Cowan et al., 2021). What is lacking is a unifying model to incorporate both the external and internal influences (Chun et al., 2011; Honey et al., 2017; Herz et al., 2020) in representing different types of memory during encoding, consolidation, and retrieval. Our study provided direct evidence for this purpose. The findings suggest that memory cue provided by task demands mainly modulates forgetting of gist and detailed information, whereas internal motivation such as interest enhances memory regardless of memory type and retention interval. In addition, including both the external and internal factors enables us to elucidate their interaction on memory and suggest a general mechanism of how memory is adaptively formed and retained by various external demands and internal needs. Neuroimaging studies have also suggested that there is a dynamic switching between brain networks in cognitive control and the default mode network for salience detection (Menon and Uddin, 2010). Future studies could clarify how these memory effects of external and internal factors are mapped into brain activity and pattern change in the hippocampus and other brain regions.

In regard to practical importance, the results suggest a possible means of decreasing the forgetting of detailed information. By providing detailed cues or instructions, participants are inclined to attend to the detailed aspects of the events, leading to slower forgetting of detailed memory (Sekeres et al., 2016; McCrudden, 2019; Staugaard and Berntsen, 2019). Thus, detailed memory cues could be adopted to improve the retention of detailed information over time. The approach could apply to a wide range of fields, such as education, clinical contexts, eyewitness testimony, and beyond. In addition, the findings extended the undermining effect to domains beyond reward and motivation (Lau et al., 2020; Murayama, 2022) and provided a practical approach to effectively employing external modulation to enhance learning and memory based on individuals’ internal states. Particularly, external cues including task demands should be cautiously used when people have higher intrinsic motivation to engage in learning and memory.

### Limitations

The current study has some limitations for future investigations. First, the number of film clips used for each condition was relatively small. Previous studies have suggested that memory could be tested by questions when naturalistic stimuli are used (e.g., Heuer and Reisberg, 1990; Furman et al., 2007, 2012; Hasson et al., 2008; Ben-Yakov and Dudai, 2011; Ben-Yakov et al., 2013, 2014). Nevertheless, the smaller number of clips may lead to insufficient power for the examination of interactions involving more than two factors. In future studies, employing a between-subject design or utilizing more materials is necessary to replicate the current results. Second, in our study, gist and detailed memories were tested by the forced-choice tasks using gist and detailed questions. Although this approach ensured that the number of gist and detailed questions were matched, it may differ from the natural recall processes in everyday life (Sekeres et al., 2016). In addition, the number of ‘remembered’ trials in our study was not sufficient for further analysis. An optimal solution would be to combine the cued-recall and forced-choice tasks in the memory tests for naturalistic events in future studies. Third, because memory cues were also used to aid the participants to retrieve specific clips, a few gist and detailed cues had overlapped information. Nevertheless, the two types of cues differ from each other in their main storylines and perceptual details. In addition, it is worth noting that a majority of the gist memory cues included action components, which may have contributed to better memory performance with gist cues. Future studies could directly manipulate this factor to clarify its contribution to memory enhancement due to gist cues.

## Conclusion

In sum, the results showed that memory cues and subjective interest differentially modulated the memory and forgetting of gist and detailed memories. Specifically, gist memory was forgotten more slowly than detailed memory when gist cues were used, while detailed memory was forgotten more slowly than gist memory when detailed cues were used. Subjective interest in the clips enhanced memory across both types of memories but did not influence the forgetting of memories. Interest also interacted with memory cue by undermining its effect when the participants were highly interested in the clips. The results provide a practical way to maintain the episodic information over a long period of time, especially for the perceptual details. Moreover, the significant interaction between the external and internal factors suggests a potential common mechanism in memory enhancement even when the external reward is not provided.

## Data availability statement

The datasets presented in this study can be found in online repositories. The names of the repository/repositories and accession number(s) can be found at: https://osf.io/smy6b.

## Ethics statement

The studies involving humans were approved by the ethics committee of School of Psychological and Cognitive Sciences, Peking University. The studies were conducted in accordance with the local legislation and institutional requirements. The participants provided their written informed consent to participate in this study.

## Author contributions

ZH designed the research, performed the research, analyzed the data, and wrote the manuscript. JY designed the research, analyzed the data, and wrote the manuscript. All authors contributed to the article and approved the submitted version.
